# Hypertension in the Ferlo (Northern Senegal): prevalence, awareness, treatment and control

**DOI:** 10.11604/pamj.2016.25.177.10105

**Published:** 2016-11-21

**Authors:** Priscilla Duboz, Gilles Boëtsch, Lamine Gueye, Enguerran Macia

**Affiliations:** 1 UMR 7268 ADèS, Téssékéré international Human-Environment Observatory, Aix-Marseille Université/EFS/CNRS, Faculté de Médecine Secteur Nord Boulevard Pierre Dramard, 13955 Marseille cedex 15, France; 2 UMI 3189 Environnement, Santé, Sociétés CNRS/Université Cheikh Anta Diop/Université de Bamako/CNRST, Laboratoire de Physiologie Exploratoire et Fonctionnelle, Faculté de Médecine de Dakar, Université Cheikh Anta Diop, Dakar, Sénégal

**Keywords:** Blood pressure, Africa, biological anthropology, awareness, treatment, control

## Abstract

**Introduction:**

The aim of this article is to assess prevalence, awareness, treatment and control of hypertension in rural Senegal (Sahelian Ferlo region).

**Methods:**

This study was carried out in 2015 on a population sample of 500 individuals living in the municipality of Tessekere, constructed using the quota method. Sociodemographic characteristics, hypertension, hypertension awareness, treatment and control, and body mass index of individuals were collected during face-to-face interviews. Statistical analyses used were Chi-square tests and binary logistic regressions.

**Results:**

Prevalence of hypertension was 31.40%. Prevalence of awareness, treatment and control among hypertensives, were 43.31%, 24.84% and 11.46% respectively. Logistic regression showed that the prevalence and awareness of hypertension increased with age. Overweight and obese subjects were more often hypertensive, but did not differ from others in awareness and treatment.

**Conclusion:**

Given the very high prevalence of hypertension in the region, a strategic approach to prevent and control hypertension is critically needed.

## Introduction

Epidemiological transition in Sub-Saharan Africa (SSA) began several years ago and is currently underway in Senegal. This transition, together with demographic transition, are the two major determinants of the increased burden of non-communicable diseases (NCD) in SSA [1, 2]. Closely linked to the progress of the epidemiological transition, hypertension is a chronic non-communicable condition of concern due to its role in the causation of coronary heart disease, stroke, and other vascular complications [3]. In 2010, hypertension in Sub-Saharan Africa was the leading risk for death, increasing by 67% since 1990 [4] and Adeloye & Basquill [5] have projected an increase to 216.8 million cases of hypertension by 2030. Moreover, stroke, the major clinical outcome of uncontrolled hypertension, has increased 46% since 1990 to become the fifth leading risk for death in SSA as well as in Senegal [4, 6]. Now, in Senegal, hypertension is rapidly becoming a major public health burden [7, 8]. The prevalence of hypertension in urban areas of this developing country has in fact already reached 27.5% [8], a rate comparable to European countries [9].

Much of the primary literature on NCD risk factors is based on studies conducted in economically advanced countries or among urban sedentary populations in Africa [10]. Few published reports exist on hypertension in rural sub-Saharan Africa, whereas the continent is still predominantly rural. In Senegal for example, 57% of the population lives in rural areas [6]. Moreover, according to Holmes et al. [11] ‘‘the assessment that hypertension is mainly a problem of cities is wrong.’’ Having studied hypertension in urban Senegal, the present study therefore sets out to analyze the prevalence of hypertension in a rural Sahelian setting populated mainly by nomadic pastoralists: the Fulani of the Ferlo, a Sahelian region in northern Senegal.

Furthermore, in SSA, despite the increasing prevalence of hypertension, rates of awareness, treatment and control remain low [8, 12–14]. Information on factors associated with awareness, treatment and control of hypertension is very scarce [15], particularly in Senegal. It is difficult to reverse the underlying pathophysiology of hypertension once it has developed. To achieve a meaningful reduction in morbidity and mortality, knowledge of the extent to which high blood pressure is being detected, treated and controlled is essential [16]. Knowing that the Fulani in the Ferlo are isolated rural populations and are poorer than urban dwellers in Senegal [17], one would expect that the Ferlo Fulani have lower rates of awareness, treatment and control than their urban counterparts.

## Methods

### Population sample

In order to carry out this study, a comprehensive survey was conducted from February to August 2015 in the municipality of Tessekere (Ferlo region, northern Senegal). In 2015, according to Senegal’s National Agency for Statistics and Demography (ANSD), a total of 8,999 individuals were living in Tessekere municipality [18]. The population sample selected for this study comprised 500 individuals aged 20 and over. The sample was constructed using the combined quota method (cross-section by age and gender) to strive for representativeness of the population of Tessekere aged 20 and over. Data from the ANSD dating from the last census (2013) were used. The quota variables used were gender (male/female) and age (20-29 / 30-39 / 40-49 / 50 and over). Practically, this method requires constructing a sample that reflects the proportions observed in the general population: for example, according to the last census, 20.6% of the population were women aged 20-29. The sample was constructed to match this proportion by including 103 women aged 20-29. The method was the same for each quota by gender, age and place of residence. Eight investigators (PhD students in Sociology, Medicine and Pharmacy) started out from different points each day to interview individuals in Wolof or Haalpulaar in every camp. Investigators had a certain number of individuals to interview (women aged 20-29 / men aged 20-29 / women aged 30-39 / men aged 30-39 / women aged 40-49 / men aged 40-49 / women aged 50 and over / men aged 50 and over) to meet the quotas. Only one person was selected as a respondent in each home. Investigators went to the house, inquired about the inhabitants and then chose the first person they saw who met the characteristics needed for the quotas. In-person interviews were conducted. They ranged from 30 to 45 minutes, depending on respondent availability and desire to talk.

### Variables studied

The selection of variables for the current analysis was hypothesis-driven based on previous literature on blood pressure awareness, treatment, and control in Africa. *Sociodemographic variables*: the socioeconomic and demographic variables collected were: age (20-29 /30-39 /40-49 /50 and over); gender (male/female); education level; defined in accordance with the educational system in Senegal; (0/1-5/6-9/10-12/over 12 years of school).

***Biological Health Variables***: the biological health variables collected were:

Blood pressure (BP): we used an OMRON M5-I digital automatic blood pressure monitor (OMRON^®^, s’Hertogenbosch, Netherlands) to take the participants’ blood pressure. Measurements were made on the upper right arm using an appropriate sized cuff while the participant was sitting and had rested for five minutes. Three readings were taken during the interview. The first was discarded, and the mean of the last two readings were used in the analysis. The first measurement was taken on both arms to detect a difference in blood pressure between arms. Hypertension was defined as a systolic BP ? 140 mmHg and/or a diastolic BP ? 90 mmHg or reported treatment for hypertension.26 Awareness of hypertension was defined as any self-reported prior diagnosis of hypertension by a health care professional among the population defined as having hypertension. Hypertensive aware participants were classified as being on treatment if they reported current use of drugs prescribed by a health professional which they had taken within the past two weeks prior to the study. Control was defined as the proportion of the sample on antihypertensive therapy with BP <140/90mmHg.

Body Mass Index: anthropometric measurements were obtained with the participant in the standing position, wearing light clothing and no footwear. Weight was measured using a digital scale (Soehnle^®^, Nassau, Germany) (measurement accuracy of 100g). To measure height, we used a Stature (Height) Measuring Stand (GPM^®^, Paris, France). Following World Health Organization recommendations, body mass index (BMI) was calculated by dividing weight (kg) by the square of the height (m²). Overweight was defined as 25 ? BMI < 30; obesity corresponded to a BMI of ? 30; underweight to a BMI of <18.5.

### Statistical analyses

Data was entered in Excel (2013), cleaned and exported to SPSS (v. 20) for analysis. The proportion of the study subjects with hypertension, subjects who were aware of their condition and who were receiving treatment was determined by sex, age group, education level and body mass index. Crude differences in these proportions across participant characteristics were determined using ?² tests. A logistic regression was then conducted to identify independent factors associated with prevalence, awareness, and treatment of hypertension. These were reported as odds ratios (ORs) with corresponding 95% confidence intervals (CIs) of hypertension prevalence, awareness and treatment (as dependent variables) after adjustment for age, sex, education level and body mass index (as independent variables). All analyses were performed using SPSS software, version 20. A p-value of < 0.05 was considered statistically significant.

## Results

Characteristics of the study sample In our 2015 survey, more than 60% of the participants were aged < 40 years. Forty-eight percent of the participants had no formal education and < 2 % had attended university (i.e. > 12 years).

### Prevalence, awareness, treatment and control of hypertension

The prevalence of hypertension was 31.40% (95% CI: 27.33% to 35.47%). Prevalence of hypertension does not differ by gender (Chi^2^ = 1.656; p = 0.198) (Table 1). However, prevalence of hypertension is significantly higher in those with no education (Chi^2^ = 8.787; p = 0.012) and among the older age brackets (Chi^2^ = 58.993; p < 0.001). In the general population, hypertension is significantly related to body mass index (Chi^2^ = 14.852; p = 0.002). In men, hypertension is only related to age (Chi^2^ = 38.659; p < 0.001) and body mass index (Chi^2^ = 13.622; p = 0.003), whereas in women, only age is significantly associated to hypertension (Chi^2^² = 39.771; p < 0.001) (Table 1).

**Table 1:** Prevalence of hypertension among women and men from Dakar according to age, education level and body mass index (N = 500)

Variables	Categories	Men				Women				Total			
		Hypertensives		Total		Hypertensives		Total		Hypertensives		Total	
		N	%	N	%	N	%	N	%	N	%	N	%
Age	20-29	18	7.47	100	41.49	17	6.56	103	39.77	35	7.00	203	40.60
	30-39	13	5.39	55	22.82	19	7.34	61	23.55	32	6.40	116	23.20
	40-49	11	4.56	35	14.52	17	6.56	42	16.22	28	5.60	77	15.40
	≥ 50	27	11.20	51	21.16	35	13.51	53	20.46	62	12.40	104	20.80
Education level	0 years	53	21.99	168	69.71	75	28.96	208	80.31	128	25.60	376	75.20
	1-5 years	12	4.98	45	18.67	13	5.0	42	16.22	25	5.00	87	17.40
	6-9 years	2	0.83	13	5.39	0	0.00	5	1.93	2	0.40	18	3.60
	10-12 years	1	0.41	11	4.56	0	0.00	2	0.77	1	0.20	13	2.60
	≥ 12 years	1	0.41	4	1.66	0	0.00	2	0.77	1	0.20	6	1.20
Body Mass Index	BMI < 18.5	15	6.22	83	34.44	20	7.72	66	25.48	35	7.00	149	29.80
	18.5 ≤ BMI < 25	42	17.43	135	56.02	42	16.22	135	52.12	84	16.80	270	54.00
	25 ≤ BMI < 30	10	4.15	21	8.71	19	7.34	45	17.37	29	5.80	66	13.20
	BMI ≥ 30	2	0.83	2	0.83	7	2.70	13	5.02	9	1.80	15	3.00
Total		69	28.63	241	100.00	88	33.98	259	100.00	157	31.40	500	100.00

^+^Education levels higher than 5 years have been aggregated in order to keep sufficient numbers for statistical tests

Among subjects with hypertension, the prevalence of awareness was 43.31% (95% CI: 31.56% to 51.06%). The general rate of awareness is higher after 50 years of age (Chi^2^ = 11.015; p = 0.001), among women (Chi^2^= 4.993; p = 0.025) and among overweight and obese subjects (Chi^2^ = 4.342; p = 0.037). No difference in hypertension awareness by education level was noticed (Chi² = 1.129; p = 0.288) (Table 2).

**Table 2:** Sociodemographic characteristics and body mass index by awareness and treatment among hypertensives (N = 157)

Variables	Categories	Not aware		Aware		Not treated		Treated		Total
		N	%	N	%	N	%	N	%	N
Sex	Men	46	51.69	23	33.82	58	49.15	11	28.21	69
	Women	43	48.31	45	66.18	60	50.85	28	71.79	88
Age	< 50	65	73.03	32	47.06	74	62.71	23	58.97	97
	≥ 50	24	26.97	36	52.94	44	37.29	16	41.03	60
Education level	0 years	70	78.65	58	85.29	97	82.20	31	79.49	128
	≥ 1 year(s)	19	21.35	10	14.71	21	17.80	8	20.51	29
Body Mass Index	BMI < 25 kg/m²	73	82.02	46	67.65	91	77.12	28	71.79	119
	BMI ≥ 25 kg/m²	16	17.98	22	32.35	27	22.88	11	28.21	38
Total		89	100.00	68	100.00	118	100.00	39	100.00	157

^+^Age brackets under 50 / education levels superior or equal to 1 year / BMIs under 25 and superior or equal to 25 kg/m² have been aggregated in order to keep sufficient numbers for statistical tests

**Figure 1:**
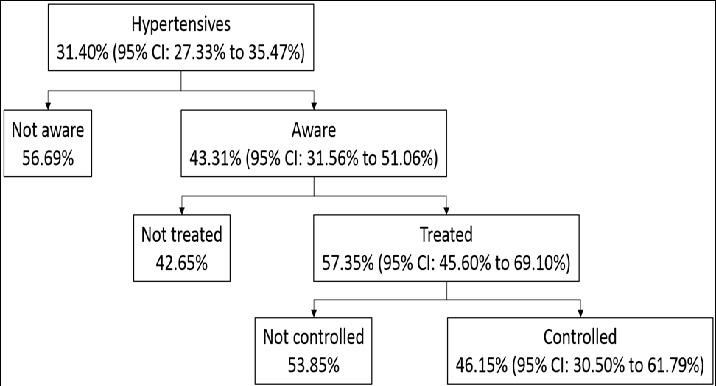
Prevalence of hypertension, awareness among hypertensives, treatment among the aware, and control among the treated in Tessekere municipality

Figure 1 shows that more than half of the hypertensive subjects who were aware of their condition were treated pharmacologically, corresponding with a prevalence of treatment among all of the hypertensives of 24.84% (95% CI: 18.08% to 31.59%). Among hypertensives, women were more likely than men to be receiving pharmacological treatment for their hypertension (Chi^2^ = 5.221; p = 0.022). However, no difference in hypertension treatment by age, body mass index and education level was noticed (Chi^2^ = 0.173; p = 0.677; Chi^2^ = 0.453; p = 0.501; Chi^2^ = 0.144; p = 0.705) (table 2).

Lastly, almost half of treated hypertensives fulfilled criteria for control, corresponding to a prevalence of control among all of the hypertensives of 11.46% (95% CI: 6.48% to 16.44%). Unfortunately, the small number of individuals with controlled BP prevented us from testing the effect of sociodemographic variables and BMI on hypertension control. The previously identified relationship between hypertension prevalence, awareness and treatment and sociodemographic variables or BMI was tested by binary logistic regression. The results of the binary logistic regressions are presented in Table 3 and Table 4.

**Table 3:** Odds ratios for prevalence of arterial hypertension by sex, age, education level and body mass index (N = 500) †

Variables	Categories	p	OR	95% CI for OR		
Sex (Men)	Women	0.558	1,13	0.74	-	1.73
Age bracket (≥ 50 years)	20-29	< 0.001+	0,16	0.09	-	0.28
	30-39	< 0.001+	0,26	0.15	-	0.47
	40-49	0.003++	0,39	0.21	-	0.72
Education level (> 12 years)	0 year	0.677	1,61	0.17	-	14.87
	1-5 years	0.600	1,83	0.19	-	17.56
	6-9 years	0.806	0,72	0.05	-	10.39
	10-12 years	0.580	0,43	0.02	-	8.77
Body mass index (≥ 25 kg/m²)	< 18.5 kg/m²	0.012^++^	0,45	0.24	-	0.84
	18.5 ≤ BMI < 25 kg/m²	0.110	0,64	0.37	-	1.11

+ < 0.001

++ < 0.05

† BMIs higher or equal to 25 kg/m² have been aggregated in order to keep sufficient numbers for statistical tests

**Table 4:** Odds ratios for awareness and treatment of arterial hypertension by sex, age, education level and body mass index (N = 157)†

Variables	Categories	Awareness					Treatment				
		p	OR	95% CI for OR			p	OR	95% CI for OR		
Sex (men)	Women	0.033^++^	2.14	1.06	-	4.28	0.023^++^	2.54	1.14	-	5.67
Age bracket (≥ 50 years)	20-49	0.003^++^	2.96	1.45	-	6.03	0.570	1.26	0.57	-	2.78
Education level (≥ 1 year(s))	0 year	0.838	0.91	0.37	-	2.23	0.446	1.46	0.55	-	3.83
Body mass index (≥ 25 kg/m²)	< 25 kg/m²	0.236	1.61	0.73	-	3.55	0.821	1.10	0.47	-	2.60

*< 0.001

**< 0.05

† Age brackets under 50 / BMIs under 25 and superior or equal to 25 kg/m² / Education level superior or equal to 1 year have been aggregated in order to keep sufficient numbers for statistical tests

Results show that actually only age and BMI were associated with hypertension prevalence. People aged 50 years old or more were more likely to have hypertension, as well as overweight or obese people (Table 3). After adjustment for age, sex, education level and BMI, the odds of hypertension awareness were higher among women and participants ? 50 years of age. Finally, in the multivariate analysis, only gender was independently associated with pharmacological treatment of hypertension (Table 4).

## Discussion

Our study is the first to address the prevalence, awareness and control of hypertension on a representative sample of Tessekere’s population. We observed a very high prevalence of hypertension. More than one-third of Tessekere adults aged 20 years and more were hypertensive, and despite the limitations of direct comparison due to methodological heterogeneity, the prevalence observed in this rural Senegalese setting is high, in the upper range of prevalence rates detected in Sub-Saharan Africa [5, 19]. Higher than that observed among other Fulani populations in Sub-Saharan Africa [20], it is comparable to the prevalence rates noted in Western countries [9]. This seems surprising, given that this population, belonging to the Fulani ethnic group and living in a harsh Sahelian environment, follows a traditional way of life (high level of physical activity related to herd movement throughout the year, a meager diet, etc.) and a low BMI [10], factors considered to prevent the onset of hypertension. However, as Campbell et al. [4] point out, *“About 30% of hypertension is attributable to increased salt consumption and about 20% is attributable to low dietary potassium (e.g., low consumption of fruits and vegetables)” and “High dietary salt is especially important in Sub Saharan Africa, as black populations are more salt sensitive, i.e., sensitive to the BP-increasing impact of sodium.”*

The Fulani populations in Senegal’s Ferlo region consume few fruits and vegetables. These factors may explain the high prevalence of hypertension among this population. As observed elsewhere, and notably in Dakar, the prevalence of hypertension increases with age [1, 16, 21–23] and body mass index [1, 15, 21, 24, 25]. We found that the prevalence of hypertension was not associated with gender, as is also the case of most African populations [1, 22, 26, 27]. Last, education level, which was associated with hypertension in ?^2^ tests, was no longer a risk factor for hypertension in logistic regression. This could be explained by the direct link between age and education level, older Tessekere inhabitants being more illiterate than younger ones.

Furthermore, the prevalence of awareness is higher than in other parts of sub-Saharan Africa, with only 56.69% of individuals unaware of their status, a result that approaches the findings obtained in Nigeria by Karaye et al. [20] in a Fulani population. This could be explained by the fact that once a year, the Ferlo’s Fulani population can access a free medical consultation, provided by a university organization. The determinants of hypertension awareness are virtually the same as those observed among an urban Senegalese population (Dakar) in 2009 [8], that is, age and gender. Indeed, as in many studies [21–23, 25, 28–30], more than one in two women and 58% of those aged 50 and over are aware of their hypertension, whereas this is the case for only one in three men and 33% of those younger than age 50. The same data are consistently found for other populations, and the hypothesis generally put forward is that women’s more frequent contact with health services through maternity and child health programs allows greater detection of hypertension during pregnancy and postpartum [22, 26, 28]. Moreover, older adults were more often aware of their hypertensive status than younger ones. A possible explanation might be that older people pay more attention to their health.

Furthermore, in this study, among hypertensives, nearly one in four people are treated. This treatment rate is comparable to that observed in a recent study of the Fulanis in Nigeria [20] and in Ghana [15]. It is higher, however, than the rate calculated for the whole of Africa [4, 31], and might be, once again, associated with the annual free medical consultation. This treatment rate is, however, much lower than European ones [23, 30] and is very far behind the 83% of hypertensives treated in the United States [25]. Moreover, the fact that hypertension treatment is associated with gender is consistent with other reports [23, 25, 28].

Lastly, the control rate note among Fulani hypertensives in the Ferlo is relatively high, particularly compared to other studies conducted on the African continent [8, 15, 28, 31], but is close to the 9.3% control rate found by Musinguzi&Nuwaha in Uganda [22]. This control rate, although high for Sub-Saharan Africa, remains low compared to the rates Wyatt et al. found in the United States in 2008. In addition, in Senegal and in most of Sub-Saharan Africa where health care is inadequate with low levels of health staffing and a chronic undersupply of medicine, achieving control of emerging NCDs such as hypertension is a virtually insurmountable task. Furthermore, infectious diseases are still prevalent in the region [6] and still represent the primary cause of mortality in Senegal, thus diminishing the possibility of taking action with regard to these public health time bombs that chronic diseases represent.

The study has some limitations. First, as in many surveys, our BP levels were based on the average of two measurements during a single visit, which may have overestimated the prevalence rates. Second, its cross-sectional design did not allow us to exclude reverse causation as the main explanation for some of the associations reported. Third, we were only able to consider pharmacological treatment. This is reductive, as lifestyle changes are a fundamental part of blood pressure control and should be considered as part of treatment. Fourth, we did not include in the study data on nutritional status and physical activity, which are major risk factors of hypertension. Finally, the small absolute number of hypertensive subjects with controlled blood pressure made it impossible to perform trend analysis on control of hypertension in age/sex subgroups.

## Conclusion

In conclusion, our study shows that 31.4% of Tessekere adults aged 20 years and more are hypertensive. The epidemiological transition currently underway in Senegal, associated with a high prevalence of HTA, also strongly affects the Senegalese rural setting examined for this study. Moreover, the prevalence observed in this rural Sub-Saharan African population is especially high, whereas individuals’ lifestyles and biometric characteristics are considered to be protective factors for hypertension; we call this the Fulani paradox. Two questions should thus be explored in the course of further studies. The first is to identify the causes of such a high prevalence of HTA in such a population (genetic or environmental causes). It is also essential to determine to what extent the use of local medicinal plants such as *Combretummicranthum* (commonly known as kinkeliba) and *Hibiscus sabdariffa* help to reduce hypertension and contribute to controlling it [32] in this area. Awareness, treatment and control rates in our study are situated in the high range for sub-Saharan Africa, and may be associated with a single free medical consultation per year. But these rates are still very low compared to those characterizing Western countries, and according to Glew et al. [10], the very low serum cholesterol concentrations of the Fulani population may predispose them to hemorrhagic stroke, especially among those who have high blood pressure. This is why, to overcome a large number of deaths among this population with a high prevalence of hypertension, it is critical to set up public health measures and intensified antihypertensive treatment to improve BP control rates and develop strategies for averting an NCD epidemic in the region.

### What is known about this topic

- Hypertension is already a major public health issue in Sub-saharan Africa, particularly in urban areas;
- Awareness, treatment and control of hypertension depends largely on people’s availability to access health care.

### What this study adds

- Hypertension is becoming a major public health issue in rural Sub-saharan Africa, notably in Fulanis;
- The prevalence observed in this rural Sub-Saharan African population is especially high, whereas individuals’ lifestyles and biometric characteristics are considered to be protective factors for hypertension.
